# Description of a new species from *Clarias maclareni* and phylogenetical analysis of *Quadriacanthus* (Monogenea, Dactylogyridae) species transfers between clariid and non-clariid fish hosts in Cameroon[Fn FN1]

**DOI:** 10.1051/parasite/2022035

**Published:** 2022-07-18

**Authors:** Dieu-Ne-Dort Bahanak, Jonathan A. Mbondo, Etienne D. Bassock Bayiha, Antoine Pariselle, Jacques Nack, Charles F. Bilong Bilong, Jean-Francois Agnèse

**Affiliations:** 1 Institute of Agricultural Research-Station for Agricultural Research of Limbe-Batoke PO Box 77 Limbe Cameroon; 2 Institute of Agricultural Research-Specialized Research Center for Marine Ecosystems PO Box 219 Kribi Cameroon; 3 Laboratory of Parasitology and Ecology, Faculty of Science, University of Yaoundé I PO Box 812 Yaoundé Cameroon; 4 ISEM, University Montpellier, CNRS, IRD 34095 Montpellier France; 5 Laboratory “Biodiversity, Ecology and Genome”, Mohammed V University in Rabat, Faculty of Science 10000 Rabat Morocco; 6 University of Douala, Faculty of Science PO Box 24157 Douala Cameroon

**Keywords:** Monogenea, *Quadriacanthus barombiensis* n. sp., Lateral transfer, Phylogeny, Africa

## Abstract

Recently in Cameroon, two species belonging to *Quadriacanthus*: *Q. anaspidoglanii* Akoumba, Tombi & Bilong Bilong, 2017 and *Q. euzeti* Nack, Pariselle & Bilong Bilong, 2016 have been recorded on gill filaments of *Notoglanidium macrostoma* (Siluriformes, Claroteidae) in the Memou’ou River (Nyong Basin) and *Papyrocranus afer* (Osteoglossiformes, Notopteridae) in Lake Ossa, respectively. These records have been considered the result of lateral transfers from Clariidae to a Claroteidae host for the first case (parasitism of *N*. *macrostoma* by *Q*. *anaspidoglanii*) and from Clariidae or Bagridae to a Notopteridae host for the second (parasitism of *P*. *afer* by *Q*. *euzeti*). In this paper, the investigation of interspecific relationships among *Quadriacanthus* spp. parasitizing Clariidae, Bagridae, Claroteidae and Notopteridae in Cameroon resulted in the record of *Q. anaspidoglanii* from *N*. *macrostoma*, *Q*. *euzeti* from *P. afer*, a new record of *Q*. *levequei* Birgi, 1988 from *Clarias jaensis* in the Nyong River, and the description of *Q*. *barombiensis* n. sp. from *Clarias maclareni* in Lake Barombi Mbo. The newly identified species is characterized by having an accessory piece ending in one small hook and the median expansion of its dorsal bar with two filaments. Phylogenetic analysis based on 28S rDNA sequences confirms that the *Quadriacanthus* spp. parasitizing gill filaments of non-clariid hosts in Cameroon originate from lateral transfers from clariid fishes, and that Clariidae are ancestral hosts of these monogenean species.

## Abstract (fr)

Récemment au Cameroun deux espèces appartenant à *Quadriacanthus* : *Q. anaspidoglanii* Akoumba, Tombi & Bilong Bilong, 2017 et *Q. euzeti* Nack, Pariselle & Bilong Bilong, 2016 ont été signalées, respectivement sur les filaments branchiaux de *Notoglanidium macrostoma* (Siluriformes, Claroteidae), dans la rivière Memou’ou (bassin du Nyong) et *Papyrocranus afer* (Osteoglossiformes, Notopteridae) dans le lac Ossa. Ces signalements ont été considérés comme le résultat de transferts latéraux de Clariidae vers un hôte Claroteidae pour le premier cas (parasitisme de *N. macrostoma* par *Q. anaspidoglanii*) et d’un Clariidae ou Bagridae vers un hôte Notopteridae pour le second (parasitisme de *P. afer* par *Q. euzeti*). Dans cet article, l’étude des relations interspécifiques entre *Quadriacanthus* spp. parasitant des Clariidae, Bagridae, Claroteidae et Notopteridae au Cameroun a abouti au signalement de *Q. anaspidoglanii* chez *N. macrostoma*, *Q. euzeti* chez *P. afer*, un nouveau signalement de *Q. levequei* Birgi, 1988 chez *Clarias jaensis* dans le fleuve Nyong, et à la description de *Q. barombiensis* n. sp. chez *Clarias maclareni* dans le lac Barombi Mbo. L’espèce nouvellement identifiée se caractérise par la présence d’une pièce accessoire se terminant par un petit crochet et l’expansion médiane de sa barre dorsale avec deux filaments. L’analyse phylogénétique basée sur les séquences d’ADNr 28S confirme que les *Quadriacanthus* spp. des filaments branchiaux parasitant des hôtes non-clariidés au Cameroun proviennent de transferts latéraux de poissons clariidés, et que les Clariidae sont bien les hôtes ancestraux de ces espèces de monogènes.

## Introduction

Monogenea have a direct life cycle. They are diversified and often host specific [12] and these characters make them an important asset to tackle the question of evolution of species or speciation [48]. As is the case for free-living organisms, speciation in parasites may occur “on-site” (sympatric/synxenic) or on vicarious sites (allopatric/alloxenic) [10]. While the second type of speciation is common for free-living organisms following migration or population isolation, it is less easy for Monogenea; in fact, being strict host specialists, they are the least probable switchers. However, once host switching succeeds, they have a high probability for speciation [7, 19, 48]. *Quadriacanthus* (Monogenea, Ancyrocephalinae) was proposed by Paperna (1961) for *Q. clariadis* Paperna, 1961 from the gills of *Clarias gariepinus* (Burchell) sampled in Israel [32]. To date 38 species are recorded in this genus from Asia and Africa [13, 45]. Although their majority (34 among the 38 known species) have been recorded from Clariid-hosts, the remaining four species have been recorded from non-clariid hosts: *Quadriacanthus bagrae* Paperna, 1979 from *Bagrus docmak* (Forsskål) and *Bagrus bajad* (Forsskål), both Bagridae [34]; *Quadriacanthus euzeti* Nack, Pariselle & Bilong Bilong, 2016 from *Papyrocranus afer* (Günther), Notopteridae [30], *Quadriacanthus anaspidoglanii* Akoumba, Tombi & Pariselle, 2017 from *Notoglanidium macrostoma* (Pellegrin), Claroteidae [2], and a fourth one, doubtful (see [17, 34]) *Quadriacanthus tilapiae* Paperna, 1973 from *Oreochromis esculentus* (Graham), Cichlidae [33]. The presence of these *Quadriacanthus* spp. on gill filaments of non-clariid hosts raises the question of their origin. The recent study by Francová *et al*. [13] on *Quadriacanthus* parasites of catfishes in eastern Africa suggests that the record of *Q*. *bagrae* on a bagriid host is the result of a lateral transfer from a clariid-host and that Clariidae are ancestral hosts of *Quadriacanthus*. In Cameroon, the presence of *Q*. *euzeti* and *Q*. *anaspidoglanii* on non-clariid fishes was also considered to originate from lateral transfers between Clariidae or Bagridae to Notopteridae for the first [30] and from Clariidae to Claroteidae for the second [2]. Because clariids, bagrids and notopterids or claroteids live in sympatry in Lake Ossa [30] and/or in the Memou’ou River [2], it was impossible, without genetic data, to determine which group was the original host family of laterally transferred *Quadriacanthus* species. Therefore, the main topic of our work concerns the use of sequence data to test the origin of these species. Taking the example of *Q*. *euzeti* in Lake Ossa, we hypothesize that if this species comes from a clariid host, it will be phylogenetically close to *Q. levequei* Birgi, 1988 (which is morphologically close to *Q*. *euzeti*) hosted by *Clarias jaensis* Boulenger in this lake [42, 43]. If *Q. euzeti* originates from a bagriid host, it will be phylogenetically close to *Q*. *bagrae* described on *B. docmak*, the sole Bagridae presently recorded in this lake. In the present study, we analyze three morphologically related *Quadriacanthus* species parasites of clariid and non-clariid fishes, namely *Q*. *levequei*, *Q*. *euzeti*, and *Q*. *anaspidoglanii* and add a new one, also morphologically similar. These four *Quadriacanthus* species are genetically compared to *Q*. *bagrae* and other *Quadriacanthus* species available in GenBank.

## Material and methods

**Figure 1:**
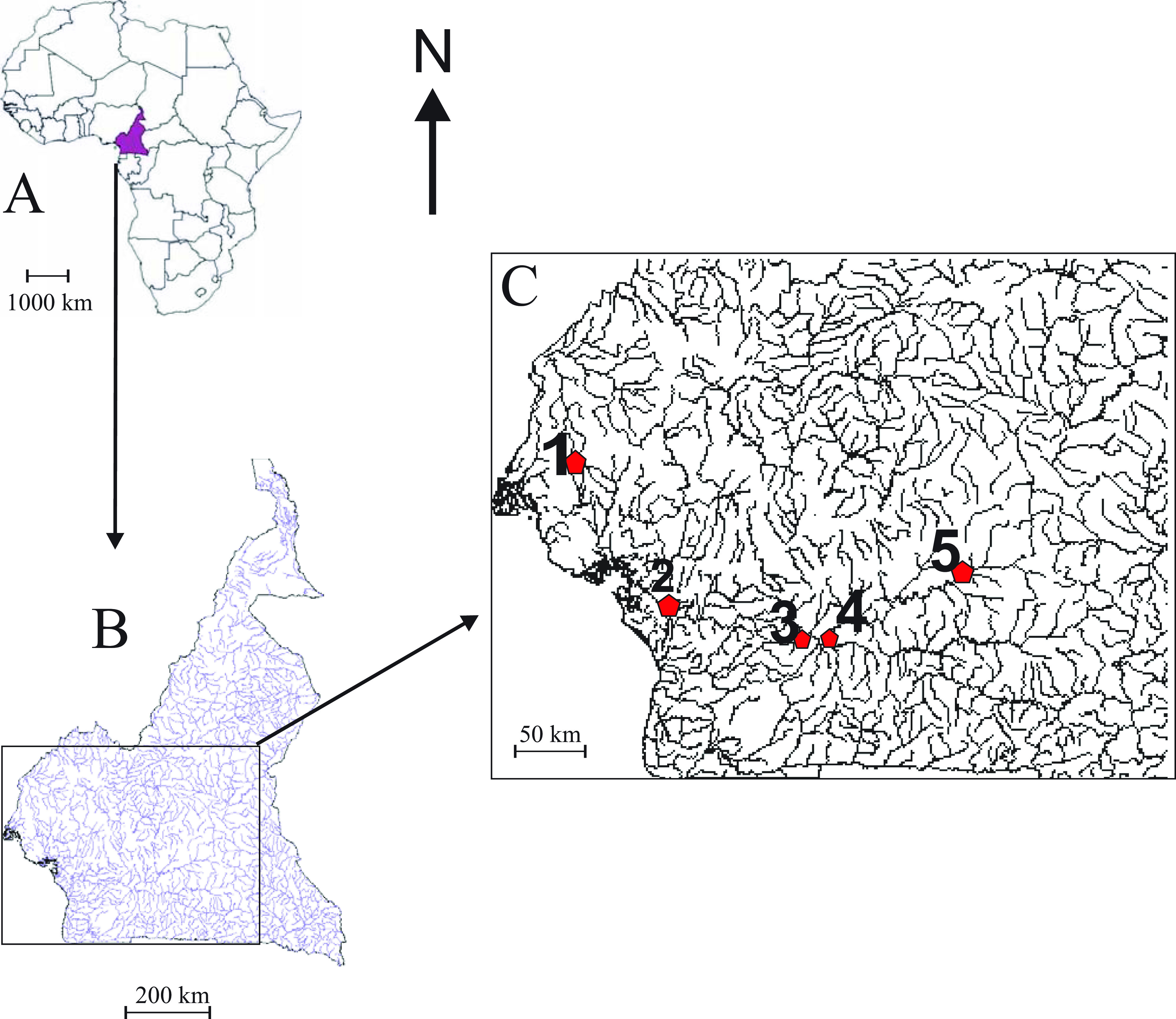
Sampling locations: (1) Lake Barombi Mbo; (2) Lake Ossa; (3, 4 & 5) Nyong River, (3) Mbalmayo market, (4) Mengong, (5) Abong Mbang. A = Africa, B = Cameroon, C = Studied area.

**Figure 2:**
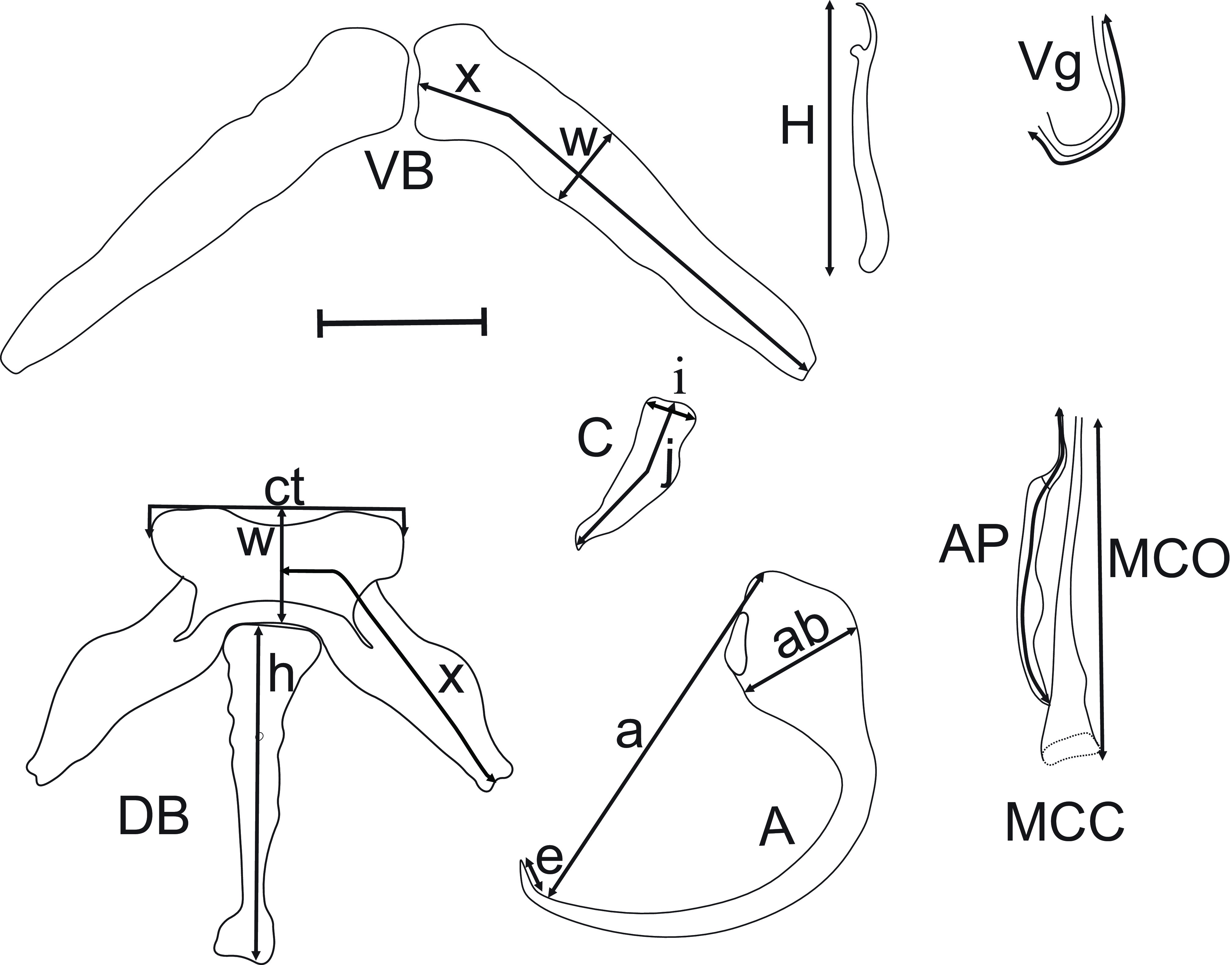
Morphometrics of *Quadriacanthus* spp. proposed by Gussev (1962) and modified by N’Douba *et al*. (1999). (A) Anchor: (*a*) length, (*ab*) base width, (*e*) point length; (Ap) Accessory piece length; (MCO) Male copulatory organ length; (C) Cuneus: (*j*) length, (*i*) width; (DB) Dorsal bar: (*ct*) center length, (*h*) median process length, (*w*) width, (*x*) length; (VB) Ventral bar: (*w*) width, (*x*) length; (H) Hook length; (Vg) Vaginal length; scale 20 µm.

Specimens of the following four species: *Clarias maclareni* Trewavas [44] (*n* = 20) endemic to Lake Barombi Mbo (4°38′ N, 9°22′ E); *C. jaensis* (*n* = 15) from the Nyong River, Mbalmayo market (3°30′48.54″ N, 11°30′04.83″ E) and Sokamalem, Abong-Mbang (03°58′21.4″ N, 13°14′53.3″ E); *A. macrostoma* (*n* = 34) from the Nyong River, Mengong (2°58′31.64″ N, 11°27′06.87″ E), and *P. afer* (*n* = 10) from Lake Ossa (4°39′ N, 9°24′ E) (Fig. 1), were caught between January 2016 to February 2017 using gill nets, cast-nets, fish-traps or hook lines, and/or purchased from fishermen. They were immediately placed in a cool box containing ice, then transported to the laboratory where they were frozen at −21 °C. In the laboratory, after thawing of the carcasses, the gill arches of fish specimens were removed by dorsal and ventral sections, then placed in a Petri-dish containing tap water. The parasites were dislodged from the gill filaments with a dissecting needle. Monogeneans were fixed individually between slide and cover slip in a drop of GAP (glycerin ammonium-picrate mixture) [22]. After 24 h, preparations were sealed using Glyceel [4]. The identification was based on the morphology and the size of sclerotized parts of the haptor and the copulatory organs. The measurements, carried out according to Gussev [14] modified by N’Douba *et al*. [27] (Fig. 2), and drawings of the sclerotized parts of the haptor and copulatory complex, were made with the aid of a Leica DM 2500 microscope, LAS software (3.8), ImageJ 1. 53 K software and Corel DrawX4^®^ software, version 14.0.0.701. Measurements, in micrometers (μm) are presented as follows: mean (minimum–maximum). Prevalence (P) and mean intensity (MI) were calculated according to Bush *et al*. [8]. Type material and vouchers were deposited in the helminth collection of the Royal Museum for Central Africa (MRAC) Tervuren (Belgium) under accession numbers MRAC 43425-43429. A principal components analysis (PCA) was performed using Statistica 6, with “standardized” measurements according to Messu *et al*. [26]. To prevent the influence of temperature or of development stage, we divided each by the length of hook II, which is supposed to keep larval size [36]. Twenty-four characters (among a total number of twenty-nine measured on each specimen, see Table 1) were retained for the PCA. Ten (10) specimens of each species included in this work were used in the PCA. For genetic purposes, fish were dissected in the field; gill arches were excised as mentioned above and stored in alcohol (95%) according to Justine *et al*. [18], then examined under stereomicroscope. Parasites found were mounted individually between slide and cover-slip in a drop of water and identified according to Birgi [6], Nack *et al*. [30] and Akoumba *et al*. [2]. After identification, each parasite was placed individually in an Eppendorf^®^ tube containing 95% alcohol. PCR was performed on these specimens according to Marchiori *et al*. [23], directly without DNA extraction. Standard PCR was performed using primers specific to the D1-D2 domain of the large subunit region (LSU) of the 28S ribosomal gene: C1 (forward; 5′ – ACCCGCTGAATTTAAGCAT – 3′) and D2 (reverse; 5′ – TGGTCCGTGTTTCAAGAC – 3′) [15]. The amplification consisted of three steps and began with 2 min at 93 °C for initial denaturation, followed by 30 cycles: 30 s at 93 °C, 30 s at 56 °C for annealing, 1 min 30 s at 72 °C for extension, with a final 5 min extension step at 72 °C. The final concentration of different reagents was as follows: GoTaq Flexibuffer (Promega) 1×, MgCl_2_ 2.5 mM, PCR nucleotide mix 0.2 nM of each DNTP, forward and reverse primers 1 μM of each, GoTaq (Promega) DNA polymerase 2 U, template DNA 0.2 μg (between 1.6 and 3 μL depending on the DNA extract concentration), nuclease-free water up to 20 μL. Sequencing was performed at the Genseq platform of ISE-M (Institute of Evolutionary Sciences of Montpellier) using the same primers as in initial PCR amplification. Purification was performed with an Agencourt^®^ AMPure^®^ PCR purification kit, following the manufacturer’s recommendations. Sequences were aligned using the Muscle program and improved manually using molecular evolutionary genetics analysis (MEGA) software [41] version 6.0. The alignments were trimmed manually using the same software. Additional 28S sequences of seven *Quadriacanthus* species namely *Quadriacanthus kobiensis* Ha, 1968 from *Clarias batrachus* (Linnaeus), *Q*. *bagrae* from *B*. *docmak*, *Q*. *mandibulatus* Francová & Řehulková, 2017 from *Heterobranchus bidorsalis* Geoffroy Saint Hilaire, *Q*. *fornicates* Francová & Řehulková, 2017, *Q*. z*uheiri* Francová & Řehulková, 2017, *Q*. *pravus* Francová & Řehulková, 2017 and *Q*. *clariadis* from *C*. *gariepinus* were retrieved for the nucleotide database GenBank (see Table 2 for accession numbers). Three species parasitizing Siluriform fish, namely *Synodontella zambezensis* Douëllou & Chishawa, 1995, *Schilbetrema* sp. and *Thaparocleidus mutabilis* (Gussev & Strelkov, 1960) and *Onchobdella aframae* Paperna, 1968 parasitizing a Cichlidae were used as the outgroup; they were obtained from GenBank. Prior to analysis, an evolutionary model was selected by MEGA 6.0 using the Bayesian information criterion (BIC). The model with the lowest BIC score was considered to better describe the pattern. Neighbor-Joining (NJ), Maximum Parsimony (MP), and Maximum Likelihood (ML) analyses were performed using MEGA version 6.0, assessing nodal support non-parametric bootstrap with 1000 replicates.

**Table 1:** Measurements of the four *Quadriacanthus* species.

Measurements	*Quadriacanthus* species							
	*Q. levequei* Birgi, 1988 (ImageJ)	*Q*. *levequei* Birgi, 1988 (present study)	*Q*. *levequei* Birgi, 1988 (original)	*Q*. *euzeti* Nack, Pariselle & Bilong Bilong, 2015 (present study)	*Q*. *euzeti* Nack, Pariselle & Bilong Bilong, 2015 (original)	*Q*. *anaspidoglanii* Akoumba, Tombi & Pariselle, 2017 (original)	*Q*. *anaspidoglanii* Akoumba, Tombi & Pariselle, 2017 (present study)	*Q*. *barombiensis* n. sp
Ph	–	39 (34–45)	–	38 (35–40)	37.5 (35–40)	–	38 (34–42)	30 (27–33)
*L*	–	624 (609–778)	(500–600)	630 (560–700)	90 (80–100)	581 (413–716)	340(240–500)	579 (410–730)
*l*	–	113 (81–167)	(150–250)	82 (70–90)	100 (90–110)	114 (82–152)	90 (70–120)	148 (102–208)
MCO	35.17	38 (32–41)	(45–50)	37.8 (36–39)	38 (36–40)	34 (30–39)	34.2 (30–39)	28 (25–29)
Ap	37.4	33 (29–38)	(30–35)	29.1 (27–31)	27 (25–28)	29 (27–30)	28.7 (27–30)	23 (20–26)
Vg	–	10 (7–13)	(30–37)	17 (16–18)	17 (16–18)	14 (13–15)	16.7 (11–20)	–
HI	11.3	15 (14–16)	(12–16)	15.9 (15–16)	16.5 (16–17)	14 (13–15)	13.9 (13–15)	15 (14–16)
II	10.8	14 (13–16)	(12–16)	15.1 (14–15)	(13–14)	13 (13–15)	13.3 (12–15)	(15–16)
III	15.1	15 (14–16)	(12–16)	16.5 (16–17)	16.5 (16–17)	14 (13–15)	14.2 (13–15)	16 (15–18)
IV	20.8	23 (22–24)	(35–39)	24 (22–26)	24.5 (23–27)	19 (18–20)	19.2 (18–20)	22 (21–22)
V	15.1	15 (14–17)	(12–16)	16.6 (16–18)	16.5 (16–17)	14 (14–15)	14.2 (13–15)	17 (16–17)
VI	15.6	15 (14–18)	(12–16)	16.8 (15–18)	16.5 (16–17)	14 (13–15)	14.4 (13–15)	17 (16–18)
VII	13.6	15 (14–17)	(12–16)	16.6 (15–18)	16.5 (16–17)	14 (14–15)	14.3 (14–15)	17 (16–18)
DB *x*	24.9	29 (27–31)	(15–20)	34.2 (31–36)	32.3 (31–33)	25 (22–27)	24.9 (22–27)	25 (24–27)
*w*	9.8	13 (11–14)	(8–12)	15.7 (14–17)	15 (14–16)	12 (10–15)	11.7 (10–15)	12 (10–14)
*h*	28.3	31 (28–35)	(18–20)	25.5 (24–28)	24 (22–27)	19 (17–21)	19.4 (17–21)	30 (27–32)
*ct*	22.1	26 (22–29)	(10–16)	27.5 (24–30)	23 (22–24)	25 (24–28)	25.2 (23–28)	23 (20–25)
DA *a*	36.2	40 (38–43)	(35–40)	50.9 (49–53)	51 (49–53)	34 (30–36)	34 (32–35)	35 (33–37)
*ab*	12.4	13 (11–13)		17.2 (15–19)	15 (13–16)	13 (12–14)	12.9 (12–14)	11 (10–12)
*e*	4.3	(4–5)	(2–4)	10.7 (9–11)	9 (8–11)	5 (4–5)	4.5 (4–5)	(3–4)
DC *i*	5.3	5 (4–7)	(4–5)	8 (7–9)	8 (7–9)	5 (4–7)	4.9 (4–6)	4 (4–5)
*j*	12.3	17 (16–18)	(12 –16)	18.6 (16–21)	18 (17–20)	14 (12–16)	14 (12–16)	12 (11–13)
VC *i*	2.7	3 (2–4)	(2–3)	6.2 (4–6)	(4–5)	3 (2–3)	2.5 (2–3)	3 (2–4)
*j*	5.4	8 (6–9)	(5–8)	12 (11–12)	11 (10–12)	8 (7–9)	7.7 (5–9)	5 (5–6)
VB *x*	42.8	54 (50–57)	(38–45)	55.7 (53–57)	53 (52–54)	41 (38–44)	40.9 (38–44)	49 (46–54)
*w*	7.6	8 (7–10)	(4–8)	11.8 (10–13)	12 (10–13)	7 (6–8).	7.2 (6–8)	7 (6–8)
VA *a*	22.3	29 (26–31)	(22–26)	36.4 (34–39)	38 (33–40)	26 (25–27)	25.8 (24–27)	22 (20–22)
*ab*	7.7	10 (9–11)		11.6 (11–12)	12 (10–13)	10 (9–11)	9.9 (9–11)	9 (8–10)
*e*	14.2	15 (13–17)	(12–14)	11.1 (9–14)	14 (11–14)	14 (13–14)	13.5 (13–14)	11 (6–14)

(Ph) Pharynx; (*L*) total body length; (*l*) body width. (MCO) Male copulatory organ length; (Ap) Accessory piece length; (Vg) Vagina length. (H) Hook length (I–VII). (DB) Dorsal bar: (*x*) length, (*w*) width, (*h*) median process length, (*ct*) center length; (DA) Dorsal anchor: (*a*) length, (*ab*) base width, (*e*) point length. (DC) Dorsal cuneus: (*j*) length, (*i*) width; (VC) Ventral cuneus: (*j*) length, (*i*) width. (VB) Ventral bar: (*x*) length, (*w*) width; (VA) Ventral anchor: (*a*) length, (*ab*) base width, (*e*) point length.

**Table 2:** List of the monogenean species used in this study, including their host, geographic location, accession numbers in GenBank, and the reference of their publication.

Parasite species	Host species	Country	Accession number	Reference
*Quadriacanthus levequei*	*Clarias jaensis*	Cameroon	ON870575	Present study
*Quadriacanthus euzeti*	*Papyrocranus afer*	Cameroon	ON870576	Present study
*Quadriacanthus barombiensis* n. sp*.*	*Clarias maclareni*	Cameroon	ON870577	Present study
*Quadriacanthus anaspidoglanii*	*Anaspidoglani*s *macrostoma*	Cameroon	ON870578	Present study
*Quadriacanthus kobiensis*	*Clarias batrachus*	China	AY841874	[11]
*Quadriacanthus bagrae*	*Bagrus docmak*	Sudan	KX685951	[13]
*Quadriacanthus clariadis*	*Clarias gariepinus*	Kenya	KX685952	[13]
*Quadriacanthus fornicatus*	*Clarias gariepinus*	Sudan	KX685953	[13]
*Quadriacanthus mandibulatus*	*Heterobranchus bidorsalis*	Kenya	KX685954	[13]
*Quadriacanthus pravus*	*Clarias gariepinus*	Sudan	KX685955	[13]
*Quadriacanthus zuheiri*	*Clarias gariepinus*	Sudan	KX685956	[13]
*Synodontella zambezensis*	*Synodontis zambezensis*	South Africa	LT220022	[39]
*Onchobdella aframae*	*Hemichromis fasciatus*	Senegal	HQ010033	[24]
*Schilbetrema* sp*.*	*Paretropius debauwi*	Aquarium in the Czech Republic, Origin West Africa	KP056243	[25]
*Thaparocleidus mutabilis*	*Silurus astus*	China	EF100550	[47]

## Results

The investigation of gill filaments of one osteoglossiform (Notopteridae) and three siluriform species, resulted in the record of four monogenean species. All recorded monogeneans were dactylogyrids, with anatomy corresponding to the diagnosis of *Quadriacanthus* given by Paperna [33], amended by Kritsky and Kulo [20] and used by Nack *et al*. [30] and Bahanak *et al*. [3]: *Q*. *euzeti* from *P*. *afer* (Prevalence = 100%, Mean Intensity = 2.5), *Q*. *anaspidoglanii* from *A*. *macrostoma* (P = 98%, MI = 3.5), *Q*. *levequei* from *C*. *jaensis* (P = 40%, MI = 1.1) and *Q*. *barombiensis* n. sp. from *C*. *maclareni* (P = 80%, MI = 4.1). Below, we present the redescription of *Q*. *levequei* due to the differences observed with the original description, and the description of *Q*. *barombiensis* n. sp. New sequences were generated from each identified species in the present study.

### *Quadriacanthus levequei* Birgi, 1988

*Type-host*: *Clarias pachynema* Boulenger, 1909.

*New host*: *Clarias jaensis* Boulenger, 1903.

*Site:* gill filaments.

*Type locality*: Mefou near Yaoundé.

*New locality*: Nyong River, Mbalmayo fish market, Cameroon (3°30′48.54″ N, 11°30′04.83″ E), Sokamalem, Abong-Mbang, Cameroon (03°58′21.4″ N, 13°14′53.3″ E).

*Material*: 10 adult worms whole-mounted in GAP.

*Voucher specimen*: MRAC 43428–43429.

**Figure 3:**
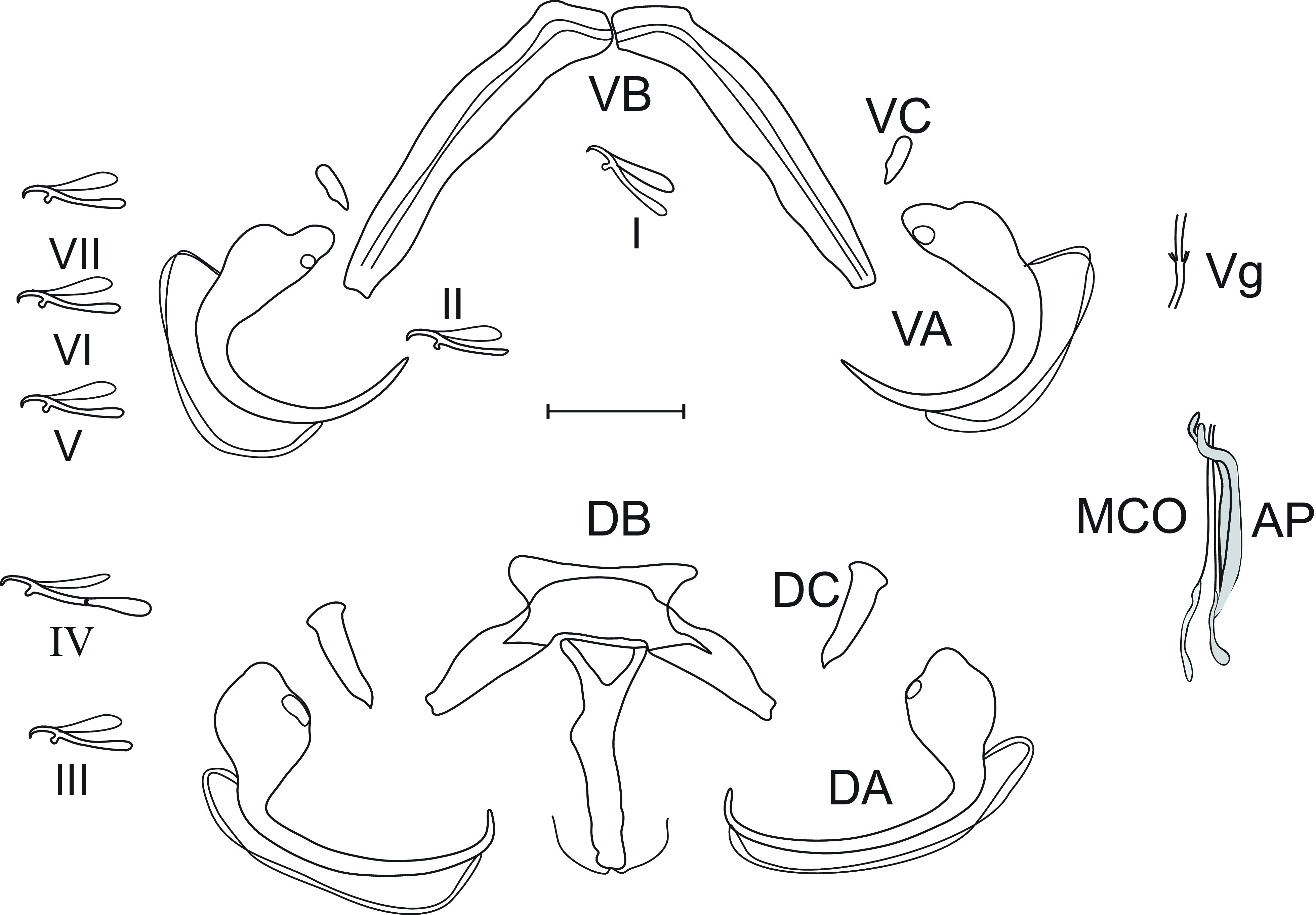
*Quadriacanthus levequei* Birgi, 1988; (VA) Ventral anchor, (DA) Dorsal anchor, (AP) Accessory piece, (MCO) Male copulatory organ, (DC) Dorsal cuneus, (VC) Ventral cuneus, (DB) Dorsal bar, (VB) Ventral bar, Hooks (I–VII), (Vg) Vagina; scale 20 μm.

*Redescription* (Fig. 3, Table 1): Adult worms 624 (609–778) long, 113 (81–167) large at the level of ovary. Pharynx circular 39 (34–45). Dorsal bar with rectangular center with two lateral expansions, one stick-shaped median process posteriorly directed with two filaments at its end. Dorsal anchors without shaft nor handle, but with regular curved blade ending with a short point. Dorsal cunei elongated. Ventral bar V-shaped made up of two medially articulated branches. Ventral anchors with shaft and handle slightly differentiated, curved blade ending with long point. Ventral cunei triangular, smaller than dorsal ones. Seven pairs of hooks, pair IV with short handle, larger than pairs I, II, III, V, VI and VII, the latter pairs about subequal. Tubular male copulatory organ (MCO) enlarged at its basal zone and tapered at distal extremity. Accessory piece straight, slightly curved distally and ending in two small, rounded hooks, one surmounting the other. Tubular vagina showing two reduced lateral expansions at its median zone.

#### Remarks

The morphology of dorsal bar with rectangular center and a median expansion stick-shaped showing two filaments at its end, the one of dorsal anchor, and the size of MCO and its accessory piece (compared to the measurements taken from the original drawings, see Table 1) of the specimens recorded in the current study on *C*. *jaensis* are similar to those of *Q. levequei* reported on *C*. *pachynema* by Birgi [6]. The differences observed between our measurements from the newly studied specimens, those taken from original drawings and those given in the original description (i.e. the size of MCO, accessory piece [AP], dorsal bar length [DB*x*], dorsal bar median process length [DB*h*], dorsal bar center length [DB*ct*], hooks pair four length [IV], Table 1) are more likely due to the different methods used to measure and draw these sclerotized parts.

### *Quadriacanthus barombiensis* n. sp. Bahanak, Nack & Pariselle (Fig. 4)

urn:lsid:zoobank.org:act:0CD26701-675B-481D-85C9-BBC4EA5C92A5

**Figure 4:**
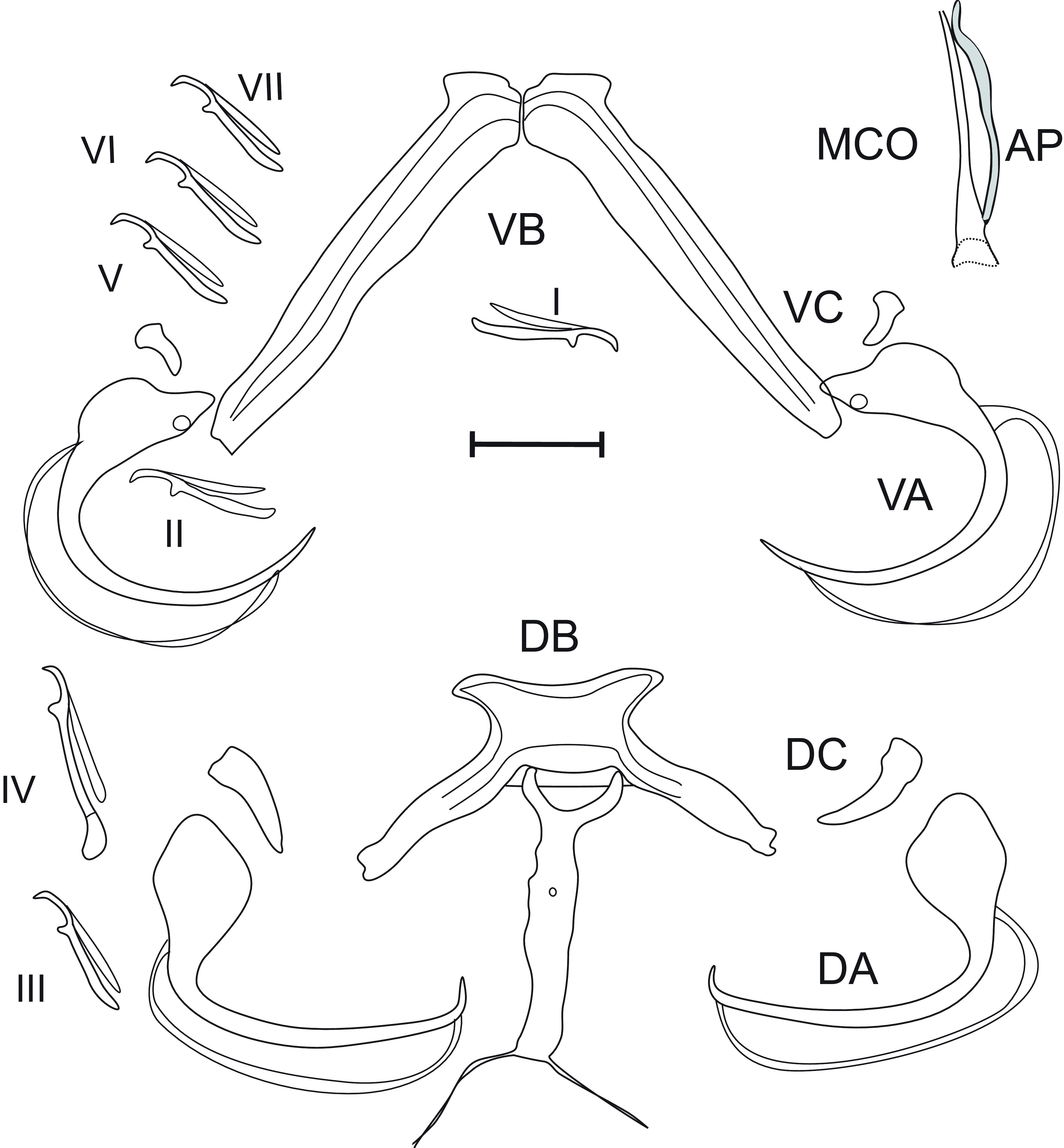
*Quadriacanthus barombiensis* n. sp. Bahanak, Nack & Pariselle; (VA) Ventral anchor, (DA) Dorsal anchor, (AP) Accessory piece, (MCO) Male copulatory organ, (DC) Dorsal cuneus, (VC) Ventral cuneus, (DB) Dorsal bar, (VB) Ventral bar, Hooks (I–VII), (Vg) Vagina; scale 20 μm.

*Type-host*: *Clarias maclareni* Trewavas, 1962.

*Site*: gill filaments.

*Type locality*: Lake Barombi Mbo, Cameroon (4°38′ N, 9°22′ E).

*Material*: 30 adult worms whole-mounted in GAP.

*Type specimens*: holotype: MRAC 43425 and paratypes: MRAC 43426–43427.

*Etymology*: Epithet *barombiensis* refers to the type locality.

*Note*: The authors of the new taxa are different from the authors of this paper: Article 50.1 and Recommendation 50A of the International Code of Zoological Nomenclature [17].

#### Description

Adult worms 579 (410–730) long, 148 (102–208) large at level of ovary. Pharynx circular 30 (27–33). Dorsal bar with rectangular center, two lateral branches, stick-shaped median process with small circular median hole, and ending with two filaments. Dorsal anchor without handle nor guard, with regular curved thin blade and short point. Ventral bar V-shaped made up of two lateral medially articulated expansions. Ventral anchor with a blade curved in an arc and ending in a long point. Ventral and dorsal cunei triangular, dorsal cuneus being larger than ventral one (see Table 1). Seven pairs of hooks, pair IV with short and pear-shaped handle, larger than pairs I, II, III, V, VI and VII, the latter pairs about subequal. Tubular MCO large at basal zone and tapered at distal extremity, accessory piece slightly S-shaped ending in one small point. Vagina not observed.

#### Remarks

By its general morphology of haptoral structures and MCO: the stick shape of dorsal bar median process (1), tubular shape of MCO enlarged at basal zone and tapered at distal end (2), and s-shape of accessory piece (3), *Q*. *barombiensis* n. sp. resembles *Q*. *levequei, Q. anaspidoglanii* and *Q*. *euzeti*; but it can easily be distinguished from its congeners by: the morphology of the distal extremity of the accessory piece with one small hook versus two small hooks in *Q. levequei* (1), the dorsal bar postero-median process with two filaments versus none in *Q*. *euzeti* and *Q*. *anaspidoglanii* (2); the vagina not sclerotized versus sclerotized in *Q*. *levequei*, *Q*. *euzeti* and *Q*. *anaspidoglanii* (3), and (4) the mean size of sclerotized parts: i.e. MCO (28 vs. 38 in *Q*. *levequei*, 34.2 in *Q*. *anaspidoglanii*, and 37.8 in *Q*. *euzeti*), accessory piece (23 vs. 33, 28.7 and 29.1), dorsal cunei (*j* = 12 vs. 17, 14 and 18.6), ventral bar (*x* = 49 vs. 54, 40.9 and 55.7).

### Principal component analysis (PCA)

**Figure 5:**
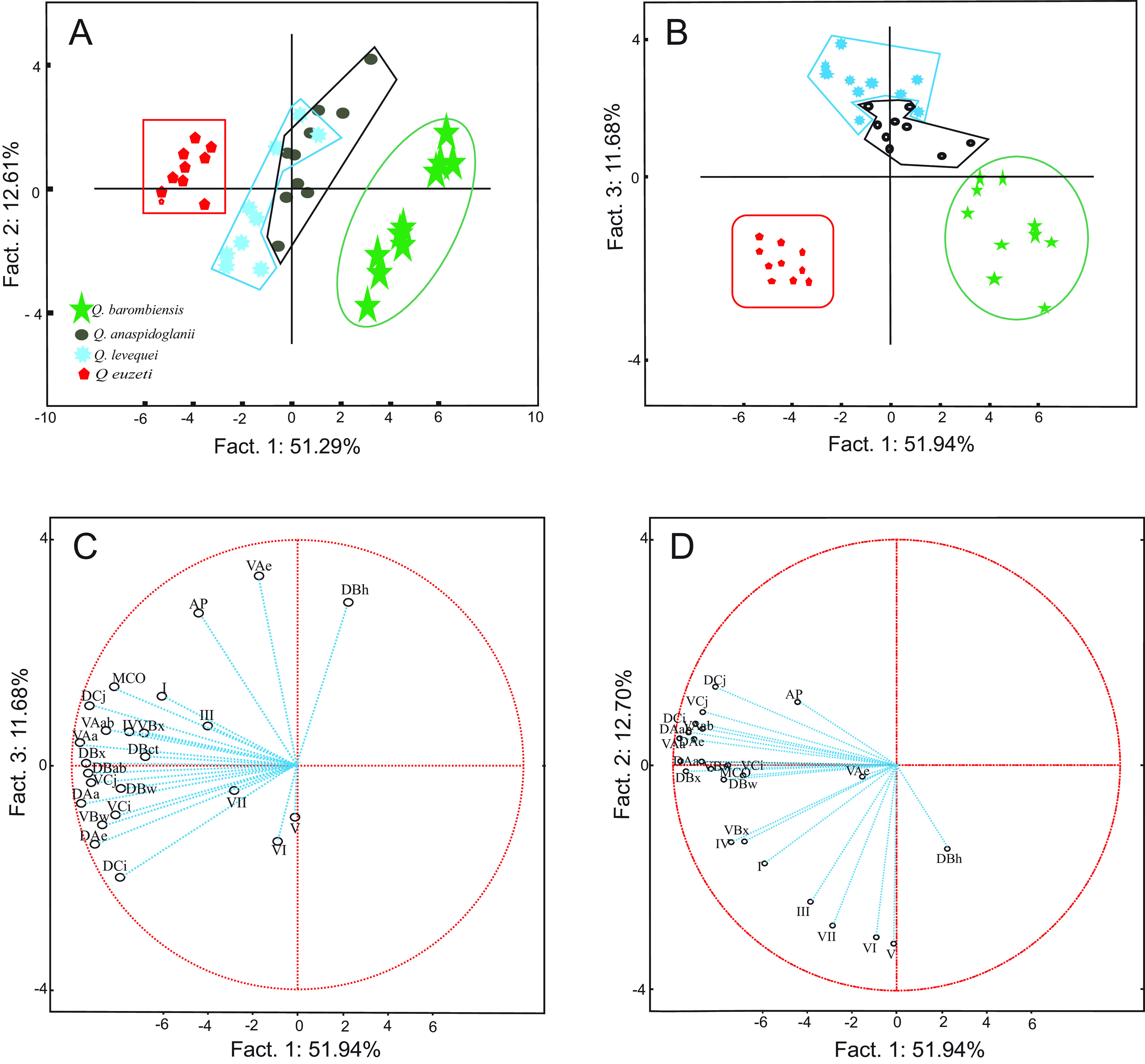
Principal component analysis scatterplot of 10 *Quadriacanthus* specimens of each of the following species: *Quadriacanthus euzeti* from *Papyrocranus afer*, *Quadriacanthus barombiensis* n. sp. from *Clarias maclareni*, *Quadriacanthus anaspidoglanii* from *Anaspidoglanis macrostoma* and *Quadriacanthus levequei* from *Clarias jaensis*. A: axes 1 and 2, B: axes 1 and 3. C and D: scatterplot of variable along axis 1 and 2 and axis 1 and 3, respectively. Dorsal anchor: (DA*a*) length, (DA*ab*) base width, (DA*e*) point length. Ventral anchor: (VA*a*) length, (VA*ab*) base width, (VA*e*) point length. (Ap) Accessory piece length, (MCO) Male copulatory organ length. Cuneus: (C*j*) length, (C*i*) width. (DB) Dorsal bar: (DB*ct*) center length, (DB*h*) median process length, (DB*w*) width, (DB*x*) length. Ventral bar: (VB*w*) width, (VB*x*) length. (I–VII) Hook length.

PCA performed on the standardized measurements of sclerotized parts of haptor and MCO of the four newly studied species, namely *Q. euzeti*, *Q*. *levequei*, *Q. anaspidoglanii* and *Q*. *barombiensis* n. sp., shows four well-defined clusters (63.90% of variance on axes 1 and 2). Specimens of *Q*. *barombiensis* n. sp. and *Q*. *euzeti* formed two isolated and clearly separated groups; however, a small overlapping zone is observed between specimens of *Q*. *anaspidoglanii* and *Q*. *levequei* (Fig. 5A). Both species are separated by axis 1 and 3 (Fig. 5B). The most represented variables and their coordinates are: ventral anchor length (VA*a* = −0.96), dorsal anchor base width (DA*ab* = −0.96), dorsal anchor length (DA*a* = −0.95), dorsal bar length (DB*x* = −0.94), dorsal cunei length (DC*j* = −0.92), ventral cunei length (VC*j* = −0.91) on axis 1; hook pair five and six length (*V* = −0.8, VI = −0.71) on axis 2 (Fig. 5C) and ventral anchor point length (VA*e* = 0.84), dorsal bar median process length (DB*h* = 0.72) on axis 3 (Fig. 5D).

### Phylogenetic analysis

**Figure 6:**
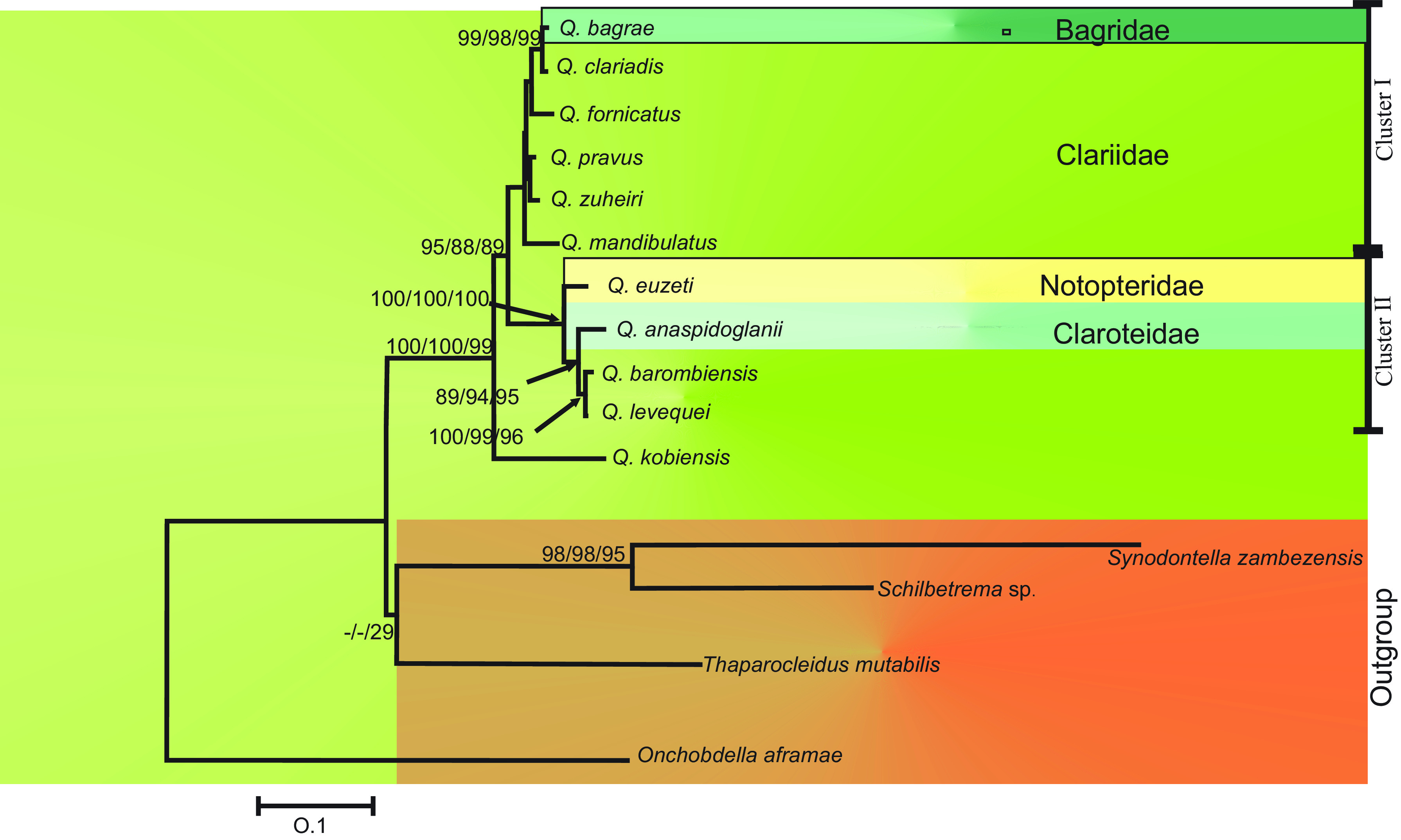
Consensus tree based on Neighbor-Joining, Maximum Parsimony and Maximum Likelihood for 28S rDNA (616 bp). Numbers indicated above the branches correspond to bootstrap values NJ/MP/ML, respectively obtained after 1000 iterations.

After trimming, the alignment of 616 positions (base pairs) was obtained, among these positions 335 variable sites were identified, 184 of which were parsimony informative. TN93 + G was selected as the best fit for our data. The analysis based on three different methods (NJ, MP and ML) produced a congruent tree topology (Fig. 6). All the *Quadriacanthus* spp. appeared clustered in one monophyletic group. *Quadriacanthus kobiensis* (Asian species) is well separated from African *Quadriacanthus* spp. and situated at the basal position of the tree. Considering African *Quadriacanthus* spp., two well-defined clusters were observed with high support. The first cluster (I) was formed by *Q*. *bagrae*, *Q*. *clariadis*, *Q*. *fornicatus*, *Q. mandibulatus*, *Q. pravus* and *Q. zuheiri* with high support. Within this cluster, *Q. bagrae* was sister species to *Q*. *clariadis* with high support. The second cluster (II) was formed by *Q*. *levequei*, *Q*. *euzeti*, *Q*. *barombiensis* and *Q*. *anaspidoglanii* with high support. Within this second cluster, *Q*. *euzeti* is separated from the other three *Quadriacanthus* spp. among which *Q*. *anaspidoglanii* was sister species to *Q*. *barombiensis* n. sp. and *Q*. *levequei*, both latter species being separated by 1% of Gamma-corrected genetic distance (Table 3, Fig. 6).

**Table 3:** Matrix of Gamma-corrected pairwise distances (in %) between 28S rDNA sequences of 616 bp length of the 15 dactylogyridean species.

		1	2	3	4	5	6	7	8	9	10	11	12	13	14
1	*Quadriacanthus levequei*														
2	*Quadriacanthus euzeti*	4.5													
3	*Quadriacanthus barombiensis* n. sp.	1.0	4.0												
4	*Quadriacanthus anaspidoglanii*	3.5	5.4	3.2											
5	*Quadriacanthus kobiensis*	14.3	14.6	14.3	13.1										
6	*Quadriacanthus bagrae*	9.0	8.1	8.6	8.6	11.9									
7	*Quadriacanthus clariadis*	8.6	8.1	8.3	8.5	12.1	1.0								
8	*Quadriacanthus fornicatus*	9.4	7.9	9.2	8.8	11.0	3.3	3.3							
9	*Quadriacanthus mandibulatus*	9.7	9.3	9.7	10.3	12.0	4.5	4.5	5.0						
10	*Quadriacanthus pravus*	7.7	7.5	7.3	7.9	11.0	2.3	2.7	3.0	3.8					
11	*Quadriacanthus zuheiri*	8.2	8.1	7.9	8.7	11.2	3.0	3.0	3.3	3.8	1.3				
12	*Synodontella zambezensis*	40.7	41.1	40.6	40.7	39.0	39.2	39.6	37.7	39.5	38.5	39.3			
13	*Onchobdella aframae*	39.8	39.5	39.7	40.2	41.3	41.6	42.2	41.7	41.9	40.6	41.3	59.3		
14	*Schilbetrema* sp.	38.2	37.2	38.3	37.8	34.9	33.8	34.0	34.2	33.5	32.7	33.7	38.1	49.0	
15	*Thaparocleidus mutabilis*	28.5	27.7	28.7	29.0	31.0	28.5	27.5	28.4	27.3	28.2	28.0	49.2	44.5	36.3

## Discussion

*Quadriacanthus barombiensis* n. sp. is specific to *C. maclareni*, i.e. oioxenous [10], as is the case for the majority of known *Quadriacanthus* species [2, 3, 6, 27–29, 30, 45]. *Quadriacanthus levequei* was previously described from the gills of *C*. *pachynema* and considered oioxenous [6]; its new record on one congeneric host (*C*. *jaensis*) changes its host specificity status from oioxenous to stenoxenous [12], even better mesostenoxenous [9]. This enlargement of the host spectrum of *Q*. *levequei* in the Nyong River Basin may have been promoted by relative phylogenetic proximity [21, 38] of *C*. *jaensis* and *C*. *pachynema* and/or local ecological conditions in the environment [30, 35]. According to Teugels [43], *C. maclareni* is morphologically close to *C. jaensis* and both species belong to sub-genus *Clarias* (*Platycephaloides*), but contrary to *C*. *jaensis* which hosts four dactylogyridean species, namely *Q*. *dageti* Birgi, 1988, *Q*. *teugelsi, Q*. *nyongensis* Birgi, 1988 and *Birgiellus calaris* Bilong Bilong, Nack and Euzet, 2007 [5], *C*. *maclareni* hosts only one species: *Q*. *barombiensis* n. sp. We assume that when the ancestor of *C*. *maclareni* colonized Lake Barombi Mbo from the Memé River system, which played a major role in fish colonization of this lake [31, 44], it could have hosted (1) several monogenean species, which have been lost due to environmental changes or following bottleneck events [37], or (2) only the ancestor of *Q*. *barombiensis* n. sp. *Clarias maclareni* being endemic in this Cameroonian volcanic line crater lake which shelters a Cichlid species flock [44], without parasite lateral transfer and/or intra-host speciation (synxenic [10]) favored by host population fragmentation, no increase of monogenean species richness has been possible [37, 46]. The close relationship between host species: *C*. *maclareni* and *C*. *jaensis* (see Teugels op. cit.), and their respective parasite species: *Q*. *barombiensis* n. sp. and *Q*. *levequei* (see Table 3 and Fig. 6), is a good illustration of a co-vicariance followed by the co-speciation of both fish and their Monogeneans [7]. *Quadriacanthus euzeti*, *Q*. *anaspidoglanii*, *Q*. *levequei*, and *Q*. *barombiensis* n. sp. (Cameroonian species, Guinean ichthyofaunan province) nested in cluster II, while *Q*. *bagrae*, *Q*. *clariadis*, *Q*. *fornicatus*, *Q*. *pravus*, *Q*. *zuheiri* and *Q*. *mandibulatus* (East African species, nilo-soudanian ichthyofaunan province) nested in cluster I. Nack *et al*. [30] did not succeed in determining whether the lateral transfer of *Q*. *euzeti* on *P. afer* (Notopteridae) originated from a Clariidae or from a Bagridae host species. The current study shows that this host switch originated from a Clariidae, presumably *C*. *jaensis*, and early took place before the speciation of other Cameroonian *Quadriacanthus* (*Q*. *anaspidoglanii*, *Q*. *levequei* and *Q*. *barombiensis* n. sp.). This type of phenomenon (transfer from a distant host family) has been recorded by Messu Mandeng *et al*. [26] in Cameroon, where *Cichlidogyrus* Paperna, 1960 usually found on cichlid hosts transferred to a cyprinodontiform host. In addition, the basal position of *Q. kobiensis*, parasite of *Clarias batrachus* (Linnaeus) from Asia (where African clariids originate [1]), suggests that members of Clariidae are ancestral hosts of *Quadriacanthus* spp. [13] and that African *Quadriacanthus* species have an Asian origin too (which was suggested by Pariselle *et al*. [37] based on the presence of additional sclerites (cunei) associated with the anchors in Asian Siluriform monogeneans and *Quadriacanthus* species). *Quadriacanthus euzeti*, *Q*. *anaspidoglanii* and *Q*. *bagrae* from non-clariid hosts are distinguished from their close congeners (*Q*. *levequei* for the two first species and *Q*. *clariadis* for *Q*. *bagrae*) hosted by clariid species, by the morphology and size of sclerotized parts of the haptor, while the copulatory organs look similar [2, 13, 20, 30, 34]: e.g. the dorsal and ventral anchor blade length, the thickness of ventral bar and the length of dorsal cunei are always reduced in *Q*. *anaspidoglanii*, while they are bigger in *Q*. *euzeti*; Francová *et al*. [13] highlight that *Q*. *bagrae* differs from *Q*. *clariadis* by the length of the ventral bar and the size of dorsal anchor blade, longer in *Q*. *clariadis*. This observation supports the adaptive nature of haptoral hard parts which are subject to selective pressure [16] such as gill morphology, encountered by these different *Quadriacanthus* spp. parasitizing distant hosts [26, 40].
